# Detecting microsatellites within genomes: significant variation among algorithms

**DOI:** 10.1186/1471-2105-8-125

**Published:** 2007-04-18

**Authors:** Sébastien Leclercq, Eric Rivals, Philippe Jarne

**Affiliations:** 1 LIRMM, UMR 5506 CNRS – Université de Montpellier II, 161 rue Ada, Montpellier, France; 2 CEFE, UMR 5175 CNRS – Université de Montpellier II, 1919 route de Mende, Montpellier, France

## Abstract

**Background:**

Microsatellites are short, tandemly-repeated DNA sequences which are widely distributed among genomes. Their structure, role and evolution can be analyzed based on exhaustive extraction from sequenced genomes. Several dedicated algorithms have been developed for this purpose. Here, we compared the detection efficiency of five of them (TRF, Mreps, Sputnik, STAR, and RepeatMasker).

**Results:**

Our analysis was first conducted on the human X chromosome, and microsatellite distributions were characterized by microsatellite number, length, and divergence from a pure motif. The algorithms work with user-defined parameters, and we demonstrate that the parameter values chosen can strongly influence microsatellite distributions. The five algorithms were then compared by fixing parameters settings, and the analysis was extended to three other genomes (*Saccharomyces cerevisiae*, *Neurospora crassa *and *Drosophila melanogaster*) spanning a wide range of size and structure. Significant differences for all characteristics of microsatellites were observed among algorithms, but not among genomes, for both perfect and imperfect microsatellites. Striking differences were detected for short microsatellites (below 20 bp), regardless of motif.

**Conclusion:**

Since the algorithm used strongly influences empirical distributions, studies analyzing microsatellite evolution based on a comparison between empirical and theoretical size distributions should therefore be considered with caution. We also discuss why a typological definition of microsatellites limits our capacity to capture their genomic distributions.

## Background

Microsatellites are genomic sequences comprised of tandem repeats of short nucleotide motifs (1 to 6 bp). They occur in all eukaryotic organisms and to a limited extent in prokaryotes, mostly in intergenic regions. Indeed, they may represent a significant part of genomes, for example about 3% of genome size (*i.e.*, millions of loci) in humans [1]. Microsatellite loci vary in length due to insertions or deletions (*i.e.*, indels) of one or more repeats, which are caused by a not-fully-understood molecular phenomenon, referred to as polymerase slippage [2,3]. A peculiarity of some loci, and the main reason for their wide use in biology, is hypermutability, with a slippage mutation rate of approximatively 0.001 mutation per locus par generation in humans [3]. Biologists have been interested in studying microsatellites for at least two reasons. First, some microsatellites are involved in molecular functions, such as recombination [4] or regulation of transcription factors [5,6]. Others, present in coding regions, are involved in neurodegenerative disorders, including Fragile X Syndrome and Huntington's disease [7], and in some forms of cancer [8]. Second, they have been widely used as molecular markers in population biology [2,9]. High mutation rates result in extensive polymorphism within populations, and most microsatellites are selectively neutral. Therefore, understanding their evolutionary dynamics, especially the effect of mutation, is important [2]. These dynamics have been studied directly by analyzing the rate and nature of mutations in pedigrees [3,10]. An alternative approach uses distributions of microsatellites extracted from large stretches of DNA or fully sequenced genomes [11-13]. Theoretical distributions based on specified models of mutation can be fitted to these empirical distributions in order to infer the most appropriate model [14-17]. Hence, by understanding the evolutionary dynamics of microsatellites, we can gain both pure and applied knowledge about molecular evolution.

Given the size of sequenced genomes, microsatellite detection requires computer programs. Moreover, microsatellites may exhibit more or less complex nucleotide sequence, since stretches of tandem repeats may be interrupted by point mutations or indels and the detection of these is not trivial. A comparison of studies based on the genomic distribution of microsatellites reveals a surprising variability in the criteria used to detect microsatellites. For example, these criteria include the minimum or maximum repeat number [14-16,18], the motif type (e.g., AC) [17], or the minimum distance between successive microsatellites [16,17]. Another aspect of this variability is the method used to detect microsatellites: either it is not mentioned or it relies on home-made, poorly explained algorithms [19,20]. This variability is likely to affect empirical distributions of microsatellites, and therefore might affect the inferred mutation parameters. In addition, this comparison also reveals that imperfections (termed interruptions), are managed differently. Such imperfections are of a few types, including single mismatches in a locus, multiple mismatches at consecutive or non-consecutive positions, the succession of different motifs (compound microsatellites), and perfect microsatellites separated by several nucleotides (interrupted microsatellites) [21]. Imperfect microsatellites are generally excluded from studies, either by decomposing imperfect loci into perfect independant subparts, or by taking into account only perfect isolated loci. Both solutions provide a biased view of reality, because imperfections result from the evolutionary process, and influence the evolutionary dynamics by restricting the slippage rate [22-24]. A more integrated view on microsatellites requires more sophisticated and dedicaded algorithms.

At least a dozen detection algorithms have been described in the literature over the last ten years and they are based on three main approaches. First, combinatorial algorithms [25-27] scan genomic sequences linearly and detect tandem repeats as sub-sequences following specific construction rules. Various rules have been proposed, but these methods guarantee exhaustive detection of all sub-sequences corresponding to the rules. The second group of methods [28-30] uses algorithms that first scan genomic sequences to detect regions that may be microsatellites under given statistical rules. These regions are then submitted to validation tests that sieve out desired sequences. This pool of sequences may not be exhaustive because some sub-sequences that could pass validation tests may not be detected by statistical tests. However, these algorithms are time-efficient, and appropriate statistical criteria insure relevant results. In the third approach, algorithms align a given motif, or library of motifs, along genomic sequences [31,32]. Regions detected as microsatellites are those whose alignment score is higher than a given threshold.

The rules leading to microsatellite detection are clearly defined for all these algorithms. However, it is likely that because they are based on different mechanisms they will detect different sets of microsatellites. Moreover, the rules upon which some of these algorithms rely are defined by parameters whose value can be set by the user (this is not true of all algorithms). Detections can also be affected by the genomic sequence under consideration because of differences among the genomes (e.g., structure, GC content, and gene composition). As far as we know, no study has been conducted to compare the relative efficiency of these approaches and to evaluate how the parameter settings of given algorithms can affect empirical microsatellite distributions. Here, we analyze the distributions of mono- to hexanucleotide microsatellites using five algorithms representative of the different classes of methods, namely Mreps [27], Sputnik [33] (first approach), TRF [29] (second approach), RepeatMasker [31], and STAR [32] (third approach). Three of them (Sputnik, TRF, and RepeatMasker) are rather widely used by biologists. These distributions were characterized by microsatellite number and size, divergence from pure microsatellites (*i.e.*, imperfection level), and genomic position. Most of the analyses were conducted using the genomic sequence of the human X chromosome, but some analyses were also conducted in three other genomes of very different size and structure (*Saccharomyces cerevisiae*, *Neurospora crassa*, and *Drosophila melanogaster*). For three algorithms (Sputnik, TRF, and Mreps), we first evaluated the influence of variable parameter settings, and then we compared the five algorithms with fixed parameter values of Sputnik, TRF, and Mreps.

## Results

### Parameter influence

**Figure 1:**
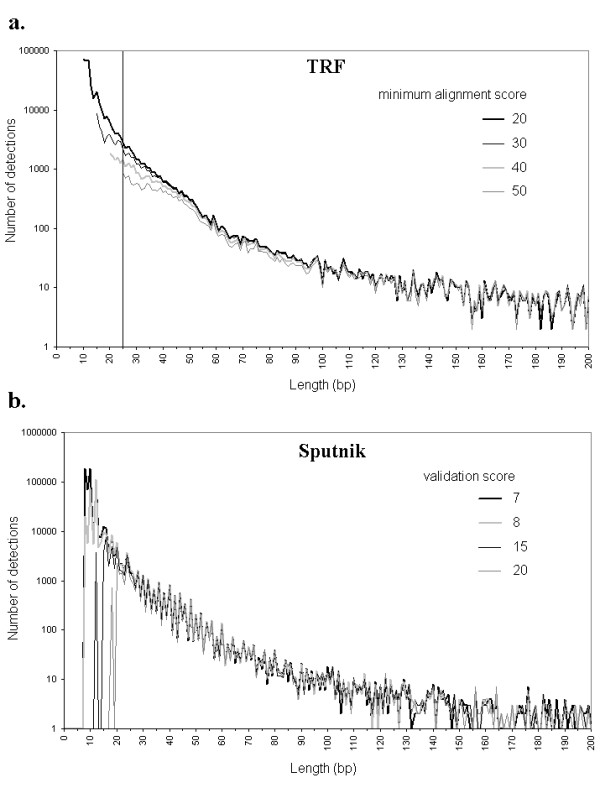
**Length distributions for different minimum threshold scores of TRF**. **a- **Number of detections (log scale) with TRF in the human X chromosome as a function of length (in bp) for minimum threshold score between 20 and 50. The alignment weights were {2,7,7}, and the few detections larger than 200 bp were discarded. The solid vertical line represents the minimum length not affected by the threshold score constraint. **b- **Number of detections (log scale) with Sputnik in the human X chromosome as a function of length (in bp) for validation score set to 7, 8, 15, and 20. The mismatch penalty was -6, and the few detections larger than 200 bp were discarded.

The number of detections with TRF increases exponentially as the alignment score decreases from 50 to 20 (default alignment weights {2,7,7}; Table 1). This increase is paralleled by an important reduction of the average length, and a more limited reduction in divergence. The variation in detection number is mainly due to the minimum size of detections, which is correlated to the score (Figure 1a). However, for microsatellites larger than 25 bp, which are not affected by the minimum size constraint, the number of detections is still significantly larger at lower score (ANCOVA on distributions in the range 25–70 bp, *F*_3,180 _= 65.2, *P *<*0.0001*). Also note in Figure 1a the approximatively exponential decrease in detection number with length regardless of score, at least for lengths of less than 50. Modifying alignment weight also affects the number of detections, though to a more limited extent (Table 1; 61% increase between {2,7,7} and {2,3,5}). Interestingly, this is related to the detection of longer (larger than 30 bp [see Additional file 1]), more divergent microsatellites. For example, the average divergence grows from about 4% to 11.3% (Table 1). Decreasing alignment penalties for different minimum scores (20 to 40) reveals the same tendancy, with an increase in average detection length and divergence [see Additional file 2]. The validation score and mismatch penalty of Sputnik have the same effect as the alignment score and weights of TRF (Table 1). The number of detections increases exponentially as the validation score decreases because the minimum size of detections decreased. However, contrary to TRF, the validation score does not affect distributions of detections that are larger than the threshold size (Figure 1b) (ANCOVA on distributions in the range 20–70 bp, *F*_3,200 _= 0.749, *P *= *0.524*). Smaller values of mismatch penalty greatly increase the average divergence (from 0,01% with a -10 penalty to 1.19% with a -5 penalty) and slightly increase the number of detections and average length (8.5% and 4% respectively). This means that microsatellites detected with a -5 penalty are essentially a set of enlarged microsatellites detected with a -10 penalty, due to better tolerance to imperfections. The influence of Mreps resolution parameter parallels that of alignment weights in TRF and mismatch penalty of Sputnik. Indeed, larger resolution values lead to larger and more divergent detections (Table 1). Between resolutions 1 and 6, the number of detections is 25% higher, while the corresponding increase for average length and average divergence are 73.4% and 114%. Again, this means that greater values of resolution essentially enlarge existing detections by allowing more errors. Examples of detections for different parameter settings of TRF, Sputnik, and Mreps are provided in Table 2.

**Table 1:** Number of detections per megabase, average length (bp), and average divergence (%) of detections for combinations of parameters in the human X chromosome.

		**number**	**length**	**divergence**
**TRF**				
**minimum score**				
	**50**	110	64.44	3.96
	**40**	202	47.65	3.68
	**30**	458	32.14	3.21
	**20**	2425	16.07	1.60
				
**align. weights**				
	**2,7,7**	110	64.44	3.96
	**2,7,5**	125	73.62	6.01
	**2,5,5**	136	76.44	7.13
	**2,3,5**	177	83.30	11.31
				
**Mreps**				
**resolution**				
	**1**	1368	22.96	12.39
	**2**	1539	28.11	18.47
	**3**	1636	32.21	22.15
	**6**	1712	39.80	26.51
				
**Sputnik**				
**minimum score**				
	**20**	154	34.55	1.13
	**15**	349	25.39	1.06
	**8**	4273	11.23	0.48
	**7**	6589	9.74	0.44
				
**Sputnik**				
**mismatch penalty**				
	**-10**	6555	9.33	0.01
	**-6**	6589	9.74	0.44
	**-5**	6818	10.12	1.19

TRF alignment weights were set to {2,7,7} when varying the minimum threshold score, and the minimum threshold score to 50 when alignment weights varied. Mreps resolution was 1, 2, 3, and 6. Sputnik mismatch penalty was set to -6 when varying the minimum threshold score, and the minimum threshold score to 7 when varying the mismatch penalty. Match bonus and fail score were always fixed to 1 and -1, respectively. Divergence is deduced from the alignment of the detected sequence with the perfectly repeated corresponding sequence of focal consensus motif:

*divergence *= (*substitutions *+ *insertions *+ *deletions*)/*alignment length*).

**Table 2:** Detection sample obtained with TRF with different alignment weights, Sputnik with different mismatch penalty, and Mreps with different resolution, in the human X chromosome.

		**start**	**end**	**divergence**	**motif**	**sequence**
**TRF**						
**alignment scores**						
						
**2,7,7**		304646	304658	0	CTCTC	CTCTCCTCTCCTC
		304696	304713	5.55	TCCTC	TCCTCTTCTCTCCTCTCC
		305863	305872	0	CCTTC	CCTTCCCTTC
						
**2,5,7**	**c**	304646	304713	18.3099	TCTCC	CTCTCCTCTCCTCCTTCTCCGCTCCCTGCACTGCCCTCCGCTCCCTCCGGTCCTCTTCTCTCCTCTCC
		305863	305872	0	TTCCC	CCTTCCCTTC
						
**2,5,5**		304646	304713	18.0556	TCTCC	CTCTCCTCTCCTCCTTCTCCGCTCCCTGCACTGCCCTCCGCTCCCTCCGGTCCTCTTCTCTCCTCTCC
	**e**	305836	305872	17.9487	TTCCC	CCCTCTCCACTTCCTTCTCTTCC**A**C**CT**CCTTCCCTTC
						
**2,3,5**	**e**	304643	304713	18.9189	CTCCT	CTGCTCTCCTCTCCTCCTTCTCCGCTCCCTGCACTGCCCTCCGCTCCCTCCGGTCCTCTTCTCTCCTCTCC
	**n**	305765	305800	25.641	CCA	CCACACCACCTCTGACGCCCACCACAGCCCCCCACC
		305836	305872	17.9487	CCCTT	CCCTCTCCACTTCCTTCTCTTCCACCTCCTTCCCTTC
						
						
**Sputnik**						
**mismatch penalty**						
						
**-10**		552928	552935	0	AG	GAGAGAGA
		552939	552948	0	AG	GAGAGAGAGA
		552954	552963	0	AAGAG	AAGAGAAGAG
		552964	552975	0	AG	AGAGAGAGAGAG
						
**-6**		552928	552935	0	AG	GAGAGAGA
		552939	552948	0	AG	GAGAGAGAGA
	**c**	552954	552975	9.09	AAGAG	AAGAGAAGAGAGAGAGAGAGAG
						
**-5**	**c**	552928	552948	9.52	AG	GAGAGAGAAAGGAGAGAGAGA
		552954	552975	9.09	AAGAG	AAGAGAAGAGAGAGAGAGAGAG
						
						
**Mreps**						
**resolution**						
						
**1**		119591	119610	20	AAT	ACAAAAAATAATAATTATAA
		119611	119628	5.56	AAAAAT	ATAAATAAAAATAAAAAT
						
**2**	**e**	119591	119615	24	AAT	ACAAAAAATAATAATTATAAATAAA
		119611	119628	5.56	AAAAAT	ATAAATAAAAATAAAAAT
						
**3**	**c**	119591	119638	33.33	A	ACAAAAAATAATAATTATAAATAAATAAAAATAAAAATTCAACTGTAA
						
**6**	**e**	119590	119638	34.69	A	TACAAAAAATAATAATTATAAATAAATAAAAATAAAAATTCAACTGTAA

Threshold alignment score of TRF was set to 20 and alignment weights varied from {2,7,7} to {2,3,5}. Sputnik mismatch penalty was set to -10, -6, and -5. Mreps resolution value varied from 1 to 6. For each detection, we report the start/end positions, divergence from a pure repeat, motif and actual sequence. Variation of detection when reducing weights is as follows: **n**: newly detected sequence; **e**: enlargement of a previous sequence; **c**: concatenation of previous sequences. New nucleotides detected by enlarging or concatenating previous sequences are underlined. The sequence at position 305765 is an example of a microsatellite detected at low values of alignment weights of TRF. It cannot be detected with alignment weights down to {2,3,5} because correct match bonuses cannot compensate for imperfection penalties. Reducing alignment weights may also enlarge detections, as shown for alignment weights {2,5,5} at position 305836. A succession of close errors (in boldface) decreases the alignment score, which falls under the threshold score for weight values larger than {2,5,5}. Reducing alignment weights also provokes concatenation, when an enlarged tandem repeat overlaps with one of its neighbors. At position 304696, two substitutions (in boldface), stops detection when alignment weights are set to {2,7,7}. With a smaller substitution penalty (5 or less), the detection is enlarged up to position 304646 and overlaps with the other detection. Reducing Sputnik mismatch penalty allows detection of larger microsatellites, by concatenating shorter, perfect ones. The two detections at position 552928 and 552939 are concatenated with a mismatch penalty of -5, because the penalty induced by two errors at position 552936 and 552938 are compensated by the second detection. A second concatenation occurs at position 552964 with a mismatch of -6. The two merged detections are not of the same motif, but the two errors induced by this difference are compensated by the matching bases with low values of mismatch penalty.

A larger resolution value for Mreps enlarges already-detected tandem repeats. In the first part of the tandem repeat at position 119591, adjacent repeats are separated by at most one error, and this part is detected at resolution 1; however repeats TAT and AAA are separated by two errors, so the second part can only be found at resolution 2 or higher. Finally, increasing resolution provokes concatenation. Detections for resolution 2 at positions 119591 and 199611 are enlarged when resolution is 3; both periods are reduced to 1 (see explanations in Methods), and the two sequences are merged.

### Comparison of algorithms for perfect detection

Algorithms were first executed on the human X chromosome with TRF theshold score set to 20, TRF alignment weights to {2,7,7}, Mreps resolution to 1, and Sputnik mismatch penalty and validation score to -6 and 7 respectively (as explained in the *Methods *section). The distribution of perfect detections was studied first. The absolute numbers of detections are critically different, with a 80-fold ratio between the two extreme values, returned by Sputnik and RepeatMasker (6228 and 76 detections per megabase respectively). TRF (1913 detections/Mb) is three times less efficient than Sputnik, while STAR and Mreps return 135 and 285 detections/Mb respectively.

**Figure 2:**
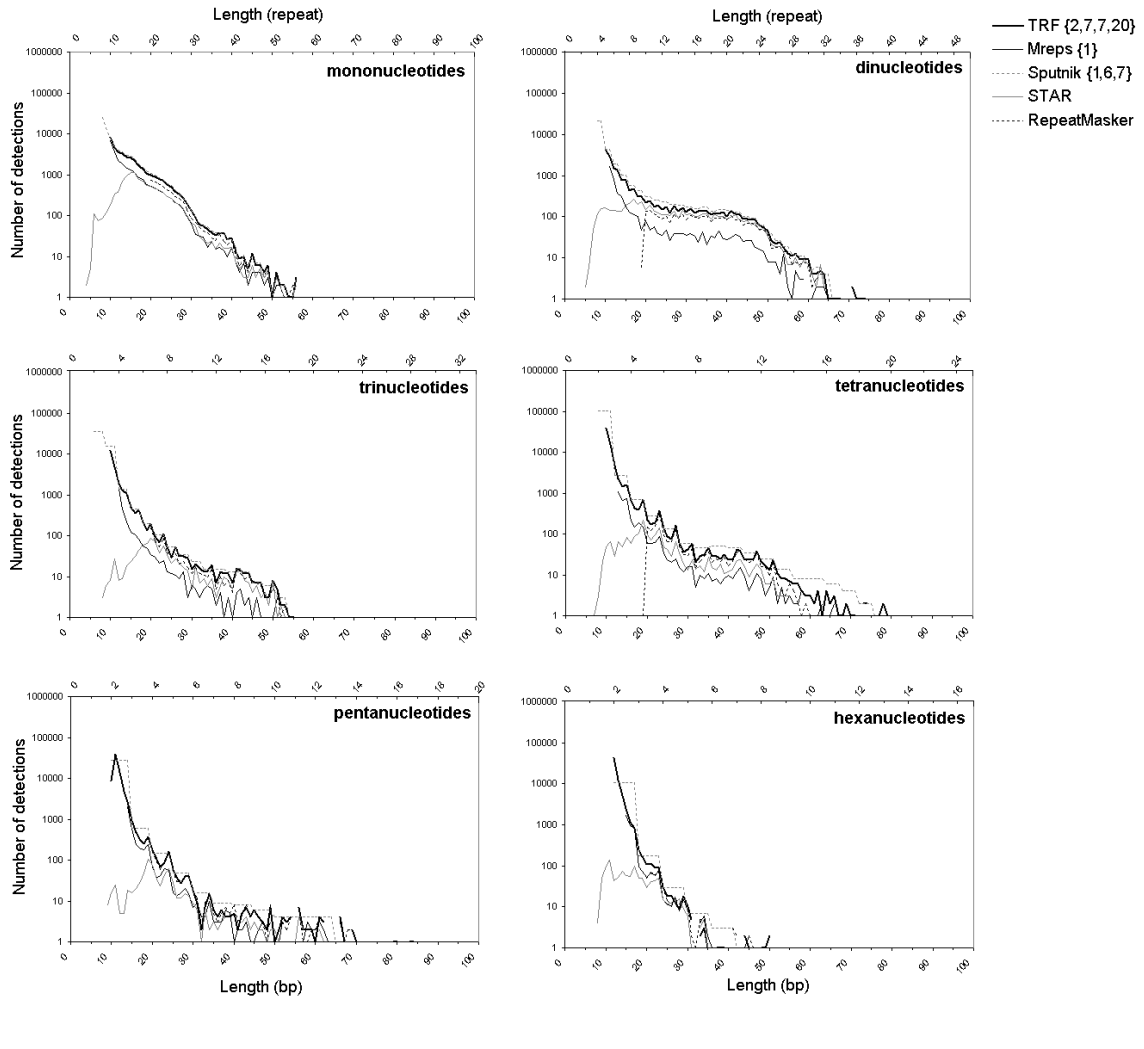
**Length distributions of perfect detections for each algorithms**. Number of perfect detections (log scale) in the human X chromosome as a function of length (in bp) for the six motif classes and for each algorithm. Sputnik groups all detections with a decimal number of repeats into the previous integer number of repeat class. The numbers of detections were averaged by motif size to display values for lengths representing a decimal number of repeats.

The comparison of length distributions revealed that the differences among algorithms depend mainly on the minimum detection length (Figure 2). For detections larger than 20 bp, the number of detections by Mreps and STAR are smaller than those of Sputnik, TRF, and RepeatMasker, for all motif classes except di- and trinucleotides (where Mreps was much less efficient). These differences are highly significant for all motif classes (ANCOVA on distributions in the range 20–70 bp, all *P *≤ 0.01), except for penta- and hexanucleotides due to a lack of power(*F*_4,50 _= 1.08, *P *= *0.376*, *F*_4,35 _= 0.223, *P *= *0.923*). It could be noticed that the 'humps' in the di- and tetranucleodite distributions previously reported [16,20] are equally detected by all algorithms. For small sizes (less than 20 bp), striking differences are observed among algorithms. First, RepeatMasker is highly constrained by its internal minimum-size threshold, which prevents detection of microsatellites that are smaller than 20 bp. On the other hand, TRF and Sputnik essentially detect microsatellites that are smaller than 15 bp for all motif classes, especially tetra- to hexanucleotides. Indeed, very short (8–12 bp) tetra- to hexanucleotides, representing detections with 2 to 2.5 repeats, are about 3.7-fold more numerous than mono- to trinucleotides of 8–12 bp (4 to 12 repeats) for TRF, and 2-fold for Sputnik. The minimum-size effect is also clearly visible with Mreps. Detection starts at 11 bp for dinucleotides, 12 bp for trinucleotides, and up to 15 bp for hexanucleotides. This explains why Mreps detects far fewer microsatellites than TRF and Sputnik. STAR distributions are very different from those returned by the three other algorithms under 20 bp, with the number of detections increasing rather than decreasing. The maximum number of detections of STAR is generally reached around 20 bp, except for dinucleotides for which the number of detections starts to decrease beyond 15 bp. Microsatellites below these sizes are at the limit to yield a local increase in compression gain. In such cases, only regions that are near enough from the previous detection are reported (see Delgrange and Rivals [32], for details).

Statistical tests were not performed for distributions of short detections (under 20 bp) because detection levels ensure critical differences.

### Comparison of algorithms for imperfect detections

Differences among algorithms for the detection of imperfect microsatellites in the human genome do not follow those observed for perfect ones. Sputnik (resp. TRF) detects only 2-fold (2.9-fold) more imperfect microsatellites than RepeatMasker, compared to the 80-fold (25-fold) ratio for perfect detections (Table 3). Moreover, Sputnik and TRF detect respectively almost 17- and 4-times less imperfect microsatellites than perfect ones, while the other algorithms detect about 2- to 4-times more imperfect than perfect microsatellites. The average length and divergence are negatively related to the number of detections for TRF, Sputnik, STAR, and RepeatMasker. For example, the highest average length and divergence are obtained for RepeatMasker, which also exhibits the lowest number of detections. The average length and number of detections are directly linked to the minimum detection length (20 bp), which prevents detection of many short microsatellites, but also increases the average divergence level (because longer microsatellites are proportionnally more imperfect; see Discussion). Similarly, high average length and divergence, and low detection number for STAR are explained by its limited capacity to detect short microsatellites (Figure 2). Interestingly, Mreps shows the reverse pattern, with the largest number of detections (1084 detections/Mb, 6-fold more than RepeatMasker) obtained for the shortest, more divergent loci.

**Table 3:** Number of detections per Mbp, average length, and average divergence for TRF, Mreps, Sputnik, STAR, and RepeatMasker, in the genome of four species.

		**HS**		**DM**	**NC**	**SC**
		**All**	**Imperfect**			
**detection number**						
	**TRF {2,7,7;20}**	2425	512	3119	2902	1822
	**Mreps 1**	1368	1084	1653	1371	879
	**Sputnik {1,-6,7}**	6589	361	7475	7665	5712
	**STAR**	395	260	311	343	182
	**RepeatMasker**	256	179	207	230	104
						
**average length**						
	**TRF {2,7,7;20}**	16.07	28.84	14.24	14.61	13.85
	**Mreps 1**	22.96	24.99	20.04	20.93	20.28
	**Sputnik {1,-6,7}**	9.74	19.83	9.39	9.35	8.98
	**STAR**	39.89	49.80	31.07	32.86	33.12
	**RepeatMasker**	53.97	64.93	48.52	45.80	54.88
						
**average divergence**						
	**TRF {2,7,7;20}**	1.60	7.59	1.61	1.47	1.35
	**Mreps 1**	12.39	15.65	11.46	10.10	11.71
	**Sputnik {1,-6,7}**	0.44	7.96	0.46	0.38	0.32
	**STAR**	7.45	11.33	7.98	6.44	7.59
	**RepeatMasker**	8.40	11.97	13.42	9.31	13.14

Both imperfect and all (perfect plus imperfect) detections are provided for the human genome while all detections only are reported for the other genomes. HS = *Homo sapiens*, SC = *Saccharomyces cerevisiae*, DM = *Drosophila melanogaster*, NC = *Neurospora crassa*. Divergence is deduced from the alignment of the detected sequence with the perfectly repeated corresponding sequence of focal consensus motif:

*divergence *= (*substitutions *+ *insertions *+ *deletions*)/*alignment length*).

When perfect and imperfect microsatellites are considered at once (Table 3), Sputnik is the most efficient in terms of the number of detections, followed by TRF and Mreps, while STAR and RepeatMasker still yield a much lower number of detections. Note also that the average size of imperfect detections is larger than the average of all detections, for all algorithms except Mreps. This confirms that imperfect and perfect microsatellites detected by Mreps have about the same length.

An important issue is whether the detections returned by the five algorithms occur at the same physical locations in genomes. This was evaluated through the 'coverage' parameter. More than 93.5% of RepeatMasker and STAR detections are also detected by Sputnik, TRF, and Mreps, with a full coverage of RepeatMasker by Sputnik (Table 4). On the other hand, the coverage of Sputnik, TRF, and Mreps by STAR and RepeatMasker is much lower (< 34% for Mreps, < 20% for TRF, and < 10% for Sputnik; Table 4). This is consistent with the fact that the latter algorithms detect more microsatellites than the former. Notably, the coverage between algorithms is also consistent with the number of detections (e.g., STAR detected 16% fewer microsatellites than TRF and 17% of the sequences detected by TRF were also detected by STAR). This suggests that detections common to the five algorithms are generally located at the same positions.

**Table 4:** Loci and nucleotide coverage between algorithms

	**B**					
		**Sputnik {1,-6,7}**	**TRF {2,7,7;20}**	**Mreps**	**STAR**	**RepeatMasker**
**A**	**Sputnik {1,-6,7}**	-	34.94 *(58.81)*	20.4 *(47.9)*	9.51 *(39.02)*	7.37 *(36.98)*
	**TRF {2,7,7;20}**	85.61 *(72.82)*	-	45.3 *(54.72)*	17.26 *(32.69)*	12.6 *(27.08)*
	**Mreps**	82.63 *(59.82)*	80.85 *(67.73)*	-	33.34 *(39.03)*	24.63 *(32.37)*
	**STAR**	95.29 *(69.56)*	93.92 *(80.03)*	93.61 *(77.31)*	-	57.98 *(66.83)*
	**RepeatMasker**	100 *(66.39)*	97.89 *(75.43)*	97.64 *(73)*	82.13 *(76.2)*	-

Proportion of the total number of detections (perfect and imperfect) of algorithm A also detected (*i.e.*, covered) by algorithm B in the human X chromosome. The value in brackets is the proportion of nucleotides detected by A and covered by B.

The coverage can also be estimated in nucleotide numbers. This method yields a slightly different answer than the one provided by the number of detections (Table 4). On the whole, frequent detections are associated with small microsatellites (Table 4; under the diagonal). The reverse pattern is observed above the diagonal of Table 4. This is again likely due to the difference in average detection sizes for the five algorithms: for example, TRF detections covered by STAR and RepeatMasker are the longest ones.

### Comparison of organisms

The algorithms were executed on three other genomic sequences and the results are presented in Table 3. The number of detections per Mbp was larger for *N. crassa*, *H. sapiens*, and *D. melanogaster *than for *S. cerevisiae*, although the difference is not significant (Kruskal-Wallis test, *H*_*observed *_= 0.85, *d.f*. = 3, *P *= *0.837*). On the other hand, a lot of variation was detected among algorithms for a given genome, as previously observed for the human X chromosome (Kruskal-Wallis test, *H*_*observed *_= 17.7, *d.f*. = 4, *P *= *0.001*). Interestingly, algorithms rank exactly in the same order for the four species with regard to the number of detections.

Comparing length and divergence also provides similar values among species for a given algorithm when considering all microsatellites (Table 3; Kruskal-Wallis tests, *H*_*observed *_= 0.337, *d.f*. = 3, *P *= *0.953 *and *H*_*observed *_= 0.577, *d.f*. = 3, *P *= *0.902 *for average length and divergence, respectively). Length distributions of perfect microsatellites in *S. cerevisiae*, *N. crassa*, and *D. melanogaster *show patterns similar to those observed in humans [see Additional file 3, 4, 5]. As for the number of detections, extensive variation is observed among algorithms for a given genome (Table 3; Kruskal-Wallis tests, *H*_*observed *_= 18.29, *d.f*. = 4, *P *= *0.001 *and *H*_*observed *_= 17.37, *d.f*. = 4, *P *= *0.002 *for average lengths and divergences, respectively). The rank order of algorithms was the same as described previously, the only exception being in *D. melanogaster *and *S. cerevisiae *where Mreps divergence is lower than that of RepeatMasker.

## Discussion and conclusion

We compared the performance of five algorithms, four of which have been developed for detecting tandem repeats. The logic underlying microsatellite detection by these five algorithms is representative of the three main approaches that are currently available (see Introduction). In order to analyze the performance of these algorithms as fully as possible, we considered several parameters (number of loci detected, length, divergence, and redundancy), the six motif lengths corresponding to the classical definition of microsatellites (mono- to hexanucleotides), and four different genomes. Our first conclusion is that in algorithms where parameter values can be modified by the user, the settings of these parameters is critical. For example, increasing TRF minimum score and Sputnik validation score allows detection of 20- to 40-times more microsatellites, especially those that are smaller and more perfect. Conversely longer and more imperfect microsatellites were detected by decreasing TRF weights, Sputnik mismatch penalty, and increasing Mreps resolution. Therefore, modifying parameter settings has important consequences.

Interestingly, this variation was not reported in the original articles [27,29] in which detection efficiency was evaluated with respect to execution time (e.g., between resolution 1 and 20 for Mreps). Delgrange and Rivals [32] noticed though the large variation in results associated with parameters setting in TRF, but were not concerned with size or divergence level. Extending our comparison to five algorithms provides generally similar results. On the whole, RepeatMasker and STAR detect fewer and longer microsatellites than TRF and Mreps (both perfect and imperfect microsatellites). Divergence is also larger for RepeatMasker and STAR than for TRF. Sputnik results are similar to those of TRF, despite a different algorithmic approach. The microsatellite sets detected by the five algorithms are also very different: on the whole, most microsatellites detected by RepeatMasker and STAR are also detected by TRF, Sputnik, and Mreps, while the reverse is far from true. Such conclusions are likely generalizable because similar results were obtained in four genomes of different sizes and GC contents. Although RepeatMasker and STAR were classified in the third approach (see Methods) while Mreps, Sputnik, and TRF are representatives of the first and second approaches, respectively, we do not conclude that the third approach generally differs in efficiency from the other two approaches.

These results require some explanation. First, the striking difference among algorithms (or even for different parameters of the same algorithm) are mainly due to differential detection of short microsatellites, especially perfect ones. The bulk of microsatellites in genomes are short (*i.e.*, less than 12 bp). More precisely, microsatellites (at least perfect ones) exhibit a negative exponential size distribution within genomes [15,16,20,34]. Large threshold sizes (e.g., with RepeatMasker, or TRF with score sets to 50) or sharp constraints on size imposed by the significance threshold (the compression gain in STAR) therefore prevents detection of the majority of microsatellites. A noteworthy contribution to these short detections by Sputnik and TRF is made by tetra-, penta-, and hexanucleotides. These microsatellites with two-to-three repeats make almost one half of the total number of microsatellites detected by TRF and they are much more numerous than expected. For example, (ACTGGT)_2 _roughly has a probability of 0.59^6 ^× 0.41^6 ^of occurence in the human genome, corresponding to about 7.3 detections on the X chromosome. TRF returned 826 detections, more than 100 times the expected value. Interestingly, the same patterns were detected in the four genomes studied. We cannot offer any clear explanation to the occurrence of these short repeats. However, even when short microsatellites are not taken into account, the five algorithms do not return the same sets of detections, therefore exhibiting different efficiencies. One reason is that the same repeat region might be interpreted differently by the five algorithms. These differences in detections are illustrated in Table 5 where some long, imperfect detections reported by RepeatMasker and STAR are decomposed into much smaller detections by Mreps (resolution 1), TRF (parameters setting {2,7,7;20}), and Sputnik (parameters setting {1,-6,7}).

**Table 5:** RepeatMasker, STAR, Mreps, TRF and Sputnik detections between starting positions 532800 and 53500 in the human X chromosome.

	**start**	**end**	**divergence**	**motif**	**sequence**
**RepeatMasker**					
	531688	531713	0	AAT	AATAATAATAATAATAATAATAATAA
	532355	532540	15.05	TTCC	TTCCTTCCTCCCTTCCTTCCTTCCTTTCTTCTTTCTTTCTTTCCTTCCTTCCTGCTTTCCTTCCTTCC
					TTTCTTTTCTTTCTTTCCTTCCTTCCTTGCTTCCTTCCTTCCATCTTTCTCTTTCTCTTTTTCTTTCT
					TTCTCTCCTTCCTTCTTTCCTTCCTTCCTTCCCTTCCCTTCCTTCCTTCC
	532704	532891	15.87	TTCC	CCTTCCTTCCTTTCTTCTTTCTTTCCTTCCTTCCTTGCTTCCTTCCTTCCATCTTTCTTTCTTTCTTT
					CTTCCTCTCCTTCCTTCTTTCCTTCCTTCCTTCCCTTCCCTCCTTCCTTTTTCTTCTTCTCTTTCTTT
					CTTTCTCTTTCCTTCCTTCCTTCCTTCTTTCTCCTTCCTTCCTTCTTTCCTT
					
**STAR**					
	531688	531713	0	AAT	AATAATAATAATAATAATAATAATAA
	532537	532731	25.38	TTTTTC	TTCCTTTTTCTTCTTCTCTTTCTTTCTTTCTTTTTCTTTCCTTCCTTCCTTCTTTCTCCTTCCTTCCT
					TCCATTTTTCTTTCTTTCTTTCTTTCTTTCTCTCTCTCTCTTTCTTTCTTTCTCTCTCTCTCTTCTTC
					CTTCCTTCCTTCCATTCTTCTTTCTTTCTTTCCTTCCTTCCTTTCTTCTTTCTTTCCTT
					
**Mreps**					
	531688	531715	3.45	AAT	AATAATAATAATAATAATAATAATAAAA
	532330	532429	15.84	TTCC	TTTCCTTCTTTCTTTCTTACTTTCTTTCCTTCCTCCCTTCCTTCCTTCCTTTCTTCTTTCTTTCTTTC
					CTTCCTTCCTGCTTTCCTTCCTTCCTTTCTTT
	532428	532467	12.5	TTCC	TTTCTTTCTTTCCTTCCTTCCTTGCTTCCTTCCTTCCATC
	532466	532490	4	TTTCTC	TCTTTCTCTTTCTCTTTTTCTTTCT
	532491	532524	11.76	TTCC	TTCTCTCCTTCCTTCTTTCCTTCCTTCCTTCCCT
	532525	532542	5.56	TTCC	TCCCTTCCTTCCTTCCTT
	532551	532593	13.95	TTTC	TCTCTTTCTTTCTTTCTTTTTCTTTCCTTCCTTCCTTCTTTCT
	532593	532609	5.88	TTCC	TCCTTCCTTCCTTCCAT
	532609	532667	16.95	TC	TTTTTCTTTCTTTCTTTCTTTCTTTCTCTCTCTCTCTTTCTTTCTTTCTCTCTCTCTCT
	532667	532689	8.7	TTCC	TTCTTCCTTCCTTCCTTCCATTC
	532690	532756	11.94	TTCC	TTCTTTCTTTCTTTCCTTCCTTCCTTTCTTCTTTCTTTCCTTCCTTCCTTGCTTCCTTCCTTCCATC
	532755	532777	4.35	TTTC	TCTTTCTTTCTTTCTTTCTTCCT
	532776	532820	8.89	TTCC	CTCTCCTTCCTTCTTTCCTTCCTTCCTTCCCTTCCCTCCTTCCTT
					
**TRF {2,7,7;20}**					
	531688	531713	0	AAT	AATAATAATAATAATAATAATAATAA
	532313	532330	5.26	TTTTC	TTTTCTTTTCTTTCTTTT
	532423	532438	5.88	TTTTC	TTTCTTTTCTTTCTTT
	532466	532490	4	TTTCTC	TCTTTCTCTTTCTCTTTTTCTTTCT
	532544	532553	0	TTC	TTCTTCTTCT
	532550	532576	13.79	TTTCTC	TTCTCTTTCTTTCTTTCTTTTTCTTTC
	532633	532667	8.57	TC	TCTCTCTCTCTCTTTCTTTCTTTCTCTCTCTCTCT
					
**Sputnik {1,-6,7}**					
	531568	531576	0	ACC	ACCACCACC
	531688	531711	0	AAT	AATAATAATAATAATAATAATAAT
	531849	531856	0	TTGC	CTTGCTTG
	531893	531900	0	TG	TGTGTGTG
	531927	531934	0	ATGC	TGCATGCA
	532078	532085	0	AGGC	GCAGGCAG
	532266	532273	0	ATGC	TGCATGCA
	532313	532322	0	TTTTC	TTTTCTTTTC
	532335	532354	5	TTTC	TTCTTTCTTTCTTACTTTCT
	532355	532422	10.29	TTCC	TTCCTTCCTCCCTTCCTTCCTTCCTTTCTTCTTTCTTTCTTTCCTTCCTTCCTGCTTTCCTTCCTTCC
	532423	532439	5.88	TTTC	TTTCTTTTCTTTCTTTC
	532440	532463	4.17	TTCC	CTTCCTTCCTTGCTTCCTTCCTTC
	532466	532489	4.17	TTTCTC	TCTTTCTCTTTCTCTTTTTCTTTC
	532500	532541	7.14	TTCC	TCCTTCTTTCCTTCCTTCCTTCCCTTCCCTTCCTTCCTTCCT
	532544	532552	0	TTC	TTCTTCTTC
	532553	532568	0	TTTC	TCTTTCTTTCTTTCTT
	532569	532576	0	TTTC	TTTCTTTC
	532577	532588	0	TTCC	CTTCCTTCCTTC
	532596	532607	0	TTCC	TTCCTTCCTTCC
	532615	532656	7.14	TTTC	TTTCTTTCTTTCTTTCTTTCTCTCTCTCTCTTTCTTTCTTTC
	532657	532666	0	TC	TCTCTCTCTC
	532669	532684	0	TTCC	CTTCCTTCCTTCCTTC
	532687	532692	0	TTC	TTCTTC
	532693	532704	0	TTTC	TTTCTTTCTTTC
	532705	532752	8.33	TTCC	CTTCCTTCCTTTCTTCTTTCTTTCCTTCCTTCCTTGCTTCCTTCCTTC
	532755	532774	0	TTTC	TCTTTCTTTCTTTCTTTCTT
	532780	532820	7.32	TTCC	CCTTCCTTCTTTCCTTCCTTCCTTCCCTTCCCTCCTTCCTT

Resolution of Mreps was set to 1, threshold alignment score of TRF to 20 and alignment weights of TRF to {2,7,7}. Sputnik mismatch penalty and validation score were set to -6 and 7, respectively. The number of detections varies with algorithms (from 3 to 18). Moreover, the sequence information is dealt with in different ways; an example is the region of cryptic simplicity between positions 532815 and 533080. RepeatMasker and STAR decompose it into large, distant and highly imperfect detections, though not the same for the two algorithms. Mreps returns a succession of shorter detections, overlapping the whole region. TRF detects only short, not much divergent, subregions, which do not completely overlap with the whole region. Sputnik detections are very numerous, short and slightly divergent, but overlap the whole region. Detection of compound microsatellites by Mreps is illustrated at position 533706, where other algorithms detect only a perfect polyA strech. The detection at position 534186 is returned as two detections by Mreps, because the two consecutive errors (insertions of G and C) stop the detection when resolution is set to 1. Very short hexanucleotides (12 bp) are detected by both TRF and Sputnik at positions 533138 and 534112. Most detections of Sputnik are two-repeat tetranucleotides, or three-repeat trinucleotides, which cannot be detected by other algorithms.

Second, Mreps detected more divergent microsatellites than the other four algorithms. This might partly be due to compound microsatellites, *i.e*, succession of motifs such as (AT)_6_(AG)_5_. Based on our definition, which considers only one motif per detection, such detections are ascribed to a single motif, here (AT)_11_.

The right part of the sequence is read as (AT)_5 _with five errors, giving a 20% divergence. Such a compound microsatellite is erroneously counted as one short imperfect detection, and would be better counted as two shorter perfect detections of different motifs. The wide average divergence is also induced by the absence of a validation score in Mreps. Such a score imposes a minimum number of correct repeats for detections to be validated. Because increasing the proportion of wrong repeats reduces the number of correct ones, detections must be longer to reach a given score. The absence of such a constraint in Mreps results in short detections that can be as divergent as long ones.

Third, the limited differences detected among the four genomes studied were not fully unexpected, though smaller than those that have been previously reported [13,15]. This result suggests that the evolution of microsatellites is related to forces that are little affected by local processes or characteristics, either genomic (e.g., GC rate, density of transposable elements) or populational (e.g., effective population size). Microsatellites are affected by two types of mutations, *i.e.*, slippage and point mutations. It might be that the net outcome of their action does not vary among genomes larger than a few tens of millions base pairs, as are those studied here.

Our results have some practical implications. First, it has become common practice, when genomes are newly sequenced, to evaluate the relative size of genomic fractions (e.g., coding *versus *non-coding sequences). Although microsatellites do not constitutes a large parts of genomes (a few percent), estimates of their density will depend on the algorithm used and on the algorithm parameters. As an example, the International Human Genome Sequencing Consortium [1] estimated that mono- to hexanucleotides constitute 1.5% of the human genome using TRF with weight {2,3,5} and score 50. This value rises to 3.9% using weight {2,7,7} and score 20, as we did here. Second, and less trivial, our results might interfere with conclusions on microsatellite evolution drawn from genomic approaches. As mentioned in Introduction, microsatellite evolution can be derived by fitting models specifying mutation parameters (e.g., slippage and point mutation rates) to actual length distributions of microsatellites. These distributions are generally built using home-made, often poorly described algorithms. For example, out of a representative set of studies following this approach [14-20,35], only two describe the algorithm used. It is therefore not clear how one can compare these approaches to the approach defined in Introduction and whether the fitted distributions are representative of the actual set of microsatellites. For those studies fitting length distributions, the extensive variation in the number of detections among algorithms might be less of an issue than the shape of the length distributions (negative exponential). However, it might be interesting to fit distributions with outcomes from different algorithms to evaluate the amount of variation in the inferred mutation processes. Another problem is that these studies focus completely on perfect microsatellites. Detecting such tandem repeats is algorithmically less complex than detecting imperfect ones, for example by scanning specific motifs and extending the search to neighbouring positions, or searching adjacent identical subsequences of the same length, whatever the motif (both approaches are combinatorial). Perfect microsatellites can also be detected using the other approaches, and filtering out *a posteriori *imperfect microsatellites. Whatsoever, we have seen that the set of returned perfect microsatellites depends on the algorithm. This, again, might affect the inference of mutational processes acting on microsatellites. Moreover, restricting the analysis to perfect microsatellites does not render the full complexity of microsatellite evolution. Note that, although some studies included imperfect microsatellites [36-38], the chosen parameters were so stringent that detection was close to perfect. These examples show an attempt to integrate imperfection into microsatellite studies, but none discuss the implications of their parameter choices, despite the non-negligible influence of imperfections on microsatellite evolutionary dynamics [22-24].

One reason why different sets of perfect microsatellites are detected by different algorithms relies on the choice of different minimum distances separating two successive microsatellites. From an algorithmic point of view, two tandemly-repeated stretches, each of the same motif, and separated by a single (or a few) nucleotide(s) (e.g., (CA)_10_G(CA)_10_) can be considered as two perfect microsatellites. From an evolutionary point of view, such a sequence is best viewed as a single imperfect microsatellite resulting from an insertion within a perfect microsatellite. A less rhetorical example can be drawn from the literature. Dieringer *et al.*, Calabrese and Durrett, and Lai and Sun [15,16,20] all looked for dinucleotides in the human genome, but used different definitions. For Lai and Sun, a detection was considered as perfect when none of the four bases on its left side were included in another detection. For Calabrese and Durrett, perfect detections must be separated by at least 50 bp and should not include a repeat of the focal motif within the 4 bp flanking sequences. Divergently, Dieringer *et al*. considered all perfect subparts as independent microsatellite detections. Counting only those detections equal to 10 repeats (from Tables and Figures in these references), the detection numbers are about 100000, 4500, and 163000 for Dieringer *et al.*, Calabrese and Durrett, and Lai and Sun respectively.

More generally, our results highlight the problem of defining a microsatellite. The simple widely-used definition is the one given in Introduction (tandem repeats of short nucleotide motifs; perfect if the same motif is repeated without interruptions, imperfect or compound otherwise [21]). However, these definitions are not precise enough to aid in decidions regarding which nucleotide regions are microsatellites. Indeed, they do not characterize the minimal required length, nor the level of imperfection. For example, compound microsatellites set specific challenges to detection methods, as mentioned above. Some attempts have been done to generalise the definition of microsatellites, for example by introducing wildcarded motifs [25]. In this case, a compound microsatellite *ATATATATACACACAC *is defined as a (A*)_8_, where * can be replaced by any nucleotide. Other authors [39] provided a first attempt to distinguish between complex and compound microsatellites, and to return them in a comprehensive way (e.g.,(AT)_4_(AC)_4_).

This typological definitions are those retained in the combinatorial algorithms used above. An important line of research would be to design new algorithms that couple microsatellite detection and the inference of the most parsimonious history of duplications and point mutations for the region being analysed [40,41]. The tandem repeat detected would then be described by both its sequence and history of duplications. In a duplication history, different motifs may be duplicated and such an approach would authorize several motifs to be involved in the formation of a single tandem repeat, as in compound microsatellites. The duplication history would help in both delimiting the tandem repeat and producing an explicit consensus sequence.

## Methods

### Algorithms

The comparative analysis was conducted using Mreps (version 2.11), Sputnik (modified version from M. Morgante 06-2001), TRF (version 3.21 for Windows), RepeatMasker (version 13-07-2002), and STAR. We will first describe at some length the logic and algorithm of these programs, because this is instrumental for understanding variation among returned microsatellite sets. In what follows, 'microsatellite' refers to those sequences we searched for, under the definitions given below. Their number, exact sequence, and positions in the genomic sequence are not known. 'Detections' are those sequences returned by algorithms. Their number is exactly known, as well as their sequence and position.

We first used Mreps which is based on the combinatorial Hamming distance algorithm for the detection of approximate repeats [42]. This algorithm considers that two adjacent sub-sequences, or repeats, with the same period (*i.e.*, repeat size) in a given sequence are part of the same tandem repeat if they differ by at most *k *mismatches. The process progresses along the sequence by comparing successive repeats and stops when two adjacent repeats differ by *k *+ 1 errors. The whole detected region is called a *k*-tandem repeat, and it is of distance *k*. For example, a perfect tandem repeat is defined by *k *= 0. Mreps searches for all possible *k*-tandem repeats with all possible periods (up to half the length of the sequence analyzed) and *k *lying between 0 and a parameter value called ŕesolution. As the Hamming Distance method stops when *k *+ 1 errors are detected, both extremities of detected regions are artefactually lengthened by erroneous nucleotides. These nucleotides are deleted by Mreps during a phase called *edge trimming*. Mreps then computes the best shortest period minimizing the average error rate of detected repeated areas (for example, transforming a periodically repeated tetranucleotide *ATAT *into a dinucleotide *AT*). The error rate is calculated as *error number/*(*length – period*), where *length *is the length of the repeated region, *period *the repeat period, and *error number *the sum of distances between all adjacent repeats. Repeats with the same best period and overlapping over at least two periods are assembled as a unique detection. Detections are filtered out in order to eliminate those expected in a random sequence, based on two filters. The *length filter *eliminates all detections smaller than *period *+ 9 bases (e.g., 11 bp for dinucleotides). The *quality filter *removes detections whose error rate and length do not satisfy internal conditions of significance. These conditions are pre-calculated by analyzing results obtained with Mreps in a pseudo-random genome, but are not detailed in Mreps documentation. Note that Mreps does not work with motifs, but with periods, so that results correspond to motif length, not to given motifs. Moreover, the Hamming distance method cannot handle indels, but this can be accounted for by using large *k *values. Indeed, indels disrupt the repeat phase, but not the repeat period. Consequently, if the distance between the two phases is smaller or equal to *k*, the two sub-sequences with different phases are considered as the same microsatellite.

The second sofware is Sputnik, which is based on a combinatorial method. Scanning the sequence from left to right, Sputnik considers that adjacent similar sub-sequences with the same period as part of the same tandem repeat. Adjacent sub-sequences are compared with the first sub-sequence of the detection. Matches increase the global score, while mismatches decrease it, and a detection is validated when reaching a threshold score. When an error decreases the score below a fail score set by the user, the comparison stops and the score is returned. Errors can be substitutions, insertions or deletions. In order to discriminate between these three possibilities, the comparison is recursively performed three times from the erroneous base. The three resulting scores are compared to the score before the erroneous position and the highest is returned. The starting position of validated detections is the first base of the first subsequence and the last position corresponds to the base associated to the best score. The algorithm resumes the procedure after this last position, for periods two to five. A post-treatment is finally applied to reduce the size of each detection to a multiple of its period.

TRF is probably the most popular algorithm for detecting tandem repeats. It was, for example, used by the International Human Genome Sequencing Consortium to detect microsatellites in the human genome sequence [1]. TRF scans sequences in order to determine regions where motifs are periodically repeated, though not necessarily tandemly repeated, based on a set of statistical rules detailed in the TRF article [29]. The most appropriate motif is then determined for each region, and this motif is aligned along the region using a Wraparound Dynamic Programming (WDP) algorithm [43]. The WDP procedure takes as input a motif and a sequence; it yields an optimal global alignment between the sequence and a perfect tandem repeat of the motif. WDP optimizes both the alignment score and the number of repeats of the motif. A score is computed from this alignment by attributing a positive weight to each correctly aligned nucleotide (*matches*), and a negative weight to substitutions (*mismatches*) and insertions-deletions (*indels or gaps*). Alignment weights can be adjusted by users, but only to a limited extend in the Windows version. When the alignment score is higher than a threshold (that can also be adjusted by the user), the alignment is returned as detection with the corresponding consensus motif. Different motifs can be aligned along a single region, in which case the three best detections only are returned. Note that the best alignment(s) might be shorter than the initially detected region.

The fourth algorithm used in this study is RepeatMasker. It was initially developed for both extracting and masking interspersed repeats from DNA sequences. As microsatellites potentially occur anywhere in genomes, they can also be considered as interspersed repeats and are searched for by RepeatMasker. However, it should be noted that RepeatMasker was not primarily developed for such a task.

RepeatMasker works with a library of reference sequences of 180 bp, each one representing the perfect repeated sequence of a given motif (e.g., (CA)_90 _or (GATA)_45_). RepeatMasker cuts the analyzed sequence in 40 Kbp pieces, overlapping over 1 Kbp. Alignment with the target sequences is based on perfect match over at least 14 bp based on the Smith-Waterman method [44]. Perfect matches separated by less than 14 bp are grouped together to constitute a single repeated region. This is conducted using the cross match program. A Smith-Waterman score is computed for the region based on predefined weights for perfect matches, substitutions, and indels. Weights are given by RepeatMasker and depend on the GC content of the 40 Kbp analyzed subsequence. The regions retained as detections are those with a Smith-Waterman score higher than a threshold (cutoff score; which can be modulated by the user). Overlapping detections are managed as follows: a detection covered over 80% of its length (or more) by another detection with a better score is not returned. RepeatMasker uses the Repbase Update Library [45] as default reference library. As some simple repeated sequences were found to be rare in the human genome, they were not included in Repbase [11]. Some penta- and hexanucleotide sequences are also missing. We therefore created a custom library containing all 501 possible reference sequences of mono- to hexanucleotide microsatellites (964 motifs with complementary ones) with sequence length set to 180 bp.

The last software we considered is STAR, which is based on a sequence-compression method, and uses the informativity of tandem repeats compared to non-repeated sequences. More specifically, STAR takes a motif as parameter, and uses a WPD algorithm [43] in order to align this motif all along the query sequence. The aligned sequence is then encoded using a lossless compression method: the encoded sequence is a succession of numbers of perfectly aligned bases (e.g., AAAAAAAAAA is encoded as 10 for motif A), and separated by encoded mismatches and indels. Good alignments lead to small encoded sequence, while the encoded sequence can be larger than the original one when the fraction of mismatches or indels is high. STAR computes a compression gain for each sequence position, as the ratio between original and encoded sequence sizes from sequence origin to this base. The gain increases in repeated regions and decreases in others. STAR uses an optimization procedure that detects the boundaries of these regions. A detection must start and end at matching positions, and series of non-matching positions could be interpreted as a non-repeated sequence between two detections, or as errors in a single detection. STAR chooses the best altenative to maximize the compression gain over the whole sequence. Algorithmic Information Theory ensures that compressible regions are significant repeated regions, which cannot be found in random sequences [46]. There is currently no statistical theory that enables one to compute the significance of an approximate tandem repeat. Thus, the rationale followed in STAR is to use the compression gain for testing the significance of a detected tandem repeat (facilitated by the Algorithmic Information Theory, also known as the Kolmogorov Complexity Theory.) and optimizing this gain globally for a set of detected tandem repeats [32]. STAR aims at finding all and only significant approximate tandem repeats of a given motif according to this criterion. STAR does not report overlapping detections because a given run focuses on one motif only, and two overlapping regions with the same motif form the same tandem repeat.

### Parameters

For these five softwares, except STAR, some input parameters are left to the user and we describe here their functions and implementations. Mreps parameters are the minimum and maximum lengths of detections (in bp), the minimum number of repeats, and the minimum and maximum motif lengths. These parameters do not affect algorithm execution, but are used to filter out final results returned to the user. Recall though that detections with a length shorter than *period *+ 9 are automatically removed (see above).

The Hamming Distance algorithm used by Mreps runs for *k*-values that are independent of the tandem repeat period. When *k *is small, large periods are penalized because few errors are allowed between adjacent repeats. Therefore, almost all sequences detected are perfect tandem repeats. On the other hand, small periods are not detected for high *k *values because only periods up to *k*+1 are searched for. The resolution parameter was implemented to bypass this problem, by running the algorithm from all values between 1 and the resolution value. This may produce overlapping detections of same periods, which are merged when they overlap over at least two periods. As a consequence, this merging step may return larger repeat regions.

Sputnik has seven standard parameters, which can be set by the user, namely the match bonus and mismatch penalty, the validation score, the fail score, the maximum number of recursions, the minimum percentage of perfection, and the period size. As for TRF, high penalty values define more stringent conditions, and the minimum detection length is directly linked to the match bonus and the validation score. Too many close errors in a row drive the score below the fail score which stops the recursion. Setting a low fail score allows merging close microsatellites with the same motif, depending on their length. The maximum number of recursions can be considered as an absolute maximum number, which stops the recursion. The minimum percentage of perfection is used, in a post-treatment filter, to discard detections not reaching this threshold. The last parameter is the period size to be searched for. We used a version of Sputnik that allowed us to search for mono- to pentanucleotides [47]. Moreover, we modified the source code to take hexanucleotides into account. In addition to these standard parameters, we used the '-j' option. By default, the first period of a detection is not counted in the score, meaning that a pentanucleotide needs to be 15 bp long to reach a score of 10, while a mononucleotide only needs to be 11 bp long. The '-j' option allows inclusion of the first repetition into the score.

In the Windows version of TRF, three parameters can be adjusted, namely the maximum motif size, alignment weights, and minimum alignment (threshold) score. The first one is a post-treament filter removing all detections with a consensus motif size larger than a given size, and takes value between 1 and 2000 bp. Alignment weights and threshold score both influence the capacity of a detected region to be validated during the scoring phase. Alignment weights include a scoring bonus (*match*) and two scoring penalties (*mismatch*, *indel*). Weights with high penalty values define more stringent conditions, because errors are more penalized during the WDP scoring computation. For example, weights list {2,7,7} is more stringent than {2,3,5} and will detect fewer imperfect microsatellites. The threshold score is the minimal score that a repeated region should reach to be validated. A high score is therefore more stringent because detections of given length must have more matching positions. Note that both the weights and threshold influence the length of detected sequences. For example, if the match bonus is +2, a score of 20 will be reached for 10 correct matches, while 25 correct matches are required to reach a score of 50. More generally, the minimum length is given by the ratio of the threshold to the match bonus.

For Repeatmasker, the cutoff value only is implemented when searching for microsatellites. It determines the minimum alignment score used by cross match to validate detections. This parameter has the same effect as the threshold score parameter of TRF: a smaller cutoff allows detecting more imperfect and/or shorter repeats, because imperfections decrease the score. However, the same cutoff value may select different sets of repeated sequences, because the scoring matrices, which are automatically chosen by RepeatMasker, depends on local GC content (see above). It is therefore difficult to evaluate how detection varies with the cutoff value. A final point is that Repeatmasker does not return detections smaller than 20 bp, independent of the cutoff value and scoring matrices.

Detection in STAR is based on differences between tandem repeats and their surrounding regions, and the complete set of information needed to run the algorithm is contained in the query sequence itself. The only information required is the type of tandem repeat, characterized by its motif. STAR does not use integrated filters based on minimum or maximum length, number of repetitions or imperfection level, and users must implement their own filters if needed.

### Redundancy

The algorithms used may detect a given tandem repeat more than once for example, when two motifs with a valid detection value represent the same sequence or when two tandem repeats overlap. Redundancy has no biological meaning and essentially results from the methods implemented by the algorithms. However, from a biological point of view, a given base in a sequence belongs to a single microsatellite. Repeatmasker partly manages redundancy by returning the detection with the highest score (see above). TRF provides detections with the three best scores. Mreps and STAR do not manage redundancy. There is no redundancy in Sputnik detections, because a new search is always initiated after the end of the previous detection. To homogenize redundancy among results, we filtered out redundant repeated areas for the four algorithms using two rules. When the shortest detection of a pair of detections overlapped the longest one by 80% or more, we kept the detection with the lowest divergence from a pure motif (defined below). In case of equal divergence, or when overlap was less than 80%, the shortest detection was discarded. When two detections overlapped over less than five nucleotides, we always kept both detections.

### Characterizing microsatellite distributions

Algorithms were compared based on five microsatellite characteristics, namely number, length, divergence compared to the consensus motif, motif class, and genomic position. As each algorithm idiosyncratically computes length and divergence depending on the detection method, we normalized definitions in order to compare algorithms. Length was defined as *end position – start position *+ 1 in bp. This was preferred to *motif length × repeat number *in order to avoid difficulties when counting indels. Divergence was defined as the number of differences between a detection and the perfectly repeated corresponding sequence of the same alignment length for the consensus motif of the detection.

*divergence *= *error number/alignment length *with *error number *= *substitutions *+ *insertions *+ *deletions*, and *alignment length *= *substitutions *+ *insertions *+ *deletions *+ *matching bases*. The algorithms used provide output values which are more or less related to divergence. Homology in TRF is the average rate of matches between adjacent repeats, based on local alignments only. Divergence could therefore not be computed from homology, and we scanned output alignment files to count both mismatches and indels. The definition of *div *in RepeatMasker differs from ours, since it provides *substitutions/*(*substitutions *+ *matching bases*). However, RepeatMasker also returns three values (*ins*, *del*, and *length*) which are defined respectively as *ins *= *insertions/*(*insertions *+ *substitutions *+ *matching bases*), *del *= *deletions/*(*deletions *+ *substitutions *+ *matching bases*), and *length *= *substitutions *+ *matching bases *+ *insertions – deletions*. Numbers of matches, substitutions, and indels were deduced from these four values. Mreps error rate (see *Algorithm *section) cannot be used to estimate divergence. A WDP algorithm [43] (see description above) was applied to Mreps detections to get number of matches, substitutions, and indels. This algorithm uses a motif as input. However, Mreps detections are returned as a succession of same period repeat units, without any consensus motif. The consensus motif was defined as the most common repeated motif in the detection. Sputnik returns a percentage of perfection as 100 × (*reference sequence length -error number*)/*reference sequence length*. This value is not compatible with our definition of divergence, so the WDP algorithm was applied to

Sputnik detections as well. STAR gives directly the number of matches, substitutions, and indels per detection. Motif classes represent the different motif sizes of microsatellites. Six classes are defined for mono- to hexanucleotides. Detections are counted in the class of its shortest period only (e.g., (AT)_12 _is counted only in class 2, and not in classes 4 or 6).

### Execution

Genome sequences depend on the evolutionary history of organisms and specific genomes may therefore vary with regard to microsatellite distribution and structure. In order to provide a general picture of the efficiency of algorithms to detect microsatellites, our study was conducted using four fully sequenced genomes spanning a range of sizes and representing very different organisms. These are the unicellular fungi *Saccharomyces cerevisiae *[48] (version Jul 26, 2004) and *Neurospora crassa OR74A *[49] (version Feb 17, 2005), the arthropod *Drosophila melanogaster *[50] (build version 4.1 Jul 21, 2005), and the vertebrate *Homo sapiens *[1] (build version 35.1, Aug 29, 2004). Genome sizes are 12 Mb (*S. cerevisiae*), 43 Mb (*N. crassa*), 110 Mb (*D. melanogaster*), and 3200 Mb (*H. sapiens*), and their average GC-content is 38%, 50%, 35%, and 41% respectively. All sequences were downloaded from the NCBI genome page [51]. Our analysis was conducted on the whole fungi sequences, but restricted to the 2L and X chromosomes of *D. melanogaster *and *H. sapiens*, respectively. Their sizes are 22 Mb and 153 Mb, but the microsatellite distributions along these chromosomes are representative of that of their whole genome (data not shown). Note also that the human, fruit fly, and *N. crassa *genomes are not fully assembled, leaving some gaps in the sequences, represented as 'N' streches. Mreps replaces gaps with random series of nucleotides, which may create artificial tandem repeats. Tandem repeats detected within gaps were excluded from the analysis.

The five programs used have default parameter values, but changing parameters may critically change length and divergence distributions as explained above. The influence of parameters on detections were first analyzed for each algorithm independently using distributions of detections from the human X chromosome. TRF default values are 500 for the maximum motif length, {2,7,7} for alignment weights, and 50 for the minimum threshold score. Microsatellites have, by definition, a motif length between 1 and 6. However, the maximum motif length was set to 10, because size 6 is not proposed in the TRF version we used. All repeats with motif size larger than 6 were discarded from the analysis. The first analysis were performed using four threshold scores (20, 30, 40, and 50) with alignment weights fixed to default. The threshold score was then fixed to default, and alignment weights to {2,7,7}, {2,5,7}, {2,5,5}, and {2,3,5}.

The default cutoff value of Repeatmasker is 225, and Smith et al. [31] suggest using values in the range 200–250 to avoid detection errors (for lower values) and underdetection (for higher values). Results obtained with different values in this window were not significantly different (data not shown), so that 225 was the cutoff value in all results reported here. Minimum and maximum motif lengths were fixed at 1 and 6 when using Mreps, as for TRF, and the minimum number of repeats was fixed at 2, representing a single tandem repeat. Mreps was run with resolution value set to 1, 2, 3, and 6. Sputnik has default parameters 1, -6, 8, and -1 for the match bonus, mismatch penalty, validation, and fail scores, respectively. The program was first executed with the validation score set to 7, 8, 10, 15, and 20. It was then set to 7, and a second analysis was performed with mismatch penalty set to -5, -6, and -10. The minimum percentage of perfection is a post-treatment filter only and does not influences the algorithm itself, so it was not investigated. The maximum-recursion parameter was evaluated, but had no influence on results for values other than 0 (which returns only perfect microsatellites). Minimum and maximum motif lengths were fixed at 1 and 6. The only input parameter in STAR is the microsatellite motif, and it was run using all 501 non-redundant, non-cyclically equivalent motifs of 1 to 6 bp long already used to construct our RepeatMasker exhaustive library.

The performance of the five algorithms was then compared. However, parameters must be adjusted for TRF and Mreps before the comparison. Rose and Falush [34] showed that the number of perfect microsatellite loci is significantly higher than expected under a random (Bernoulli) model for lengths larger or equal to 8 bp. Parameters were fixed to return microsatellites larger than 8 bp. TRF and Sputnik do not have minimal length parameter, but the threshold score restricts the minimal size of detection. A minimal length of 8 bp requires a minimum threshold score of 16 for TRF and 7 for Sputnik (because the score must be stricly larger than the threshold for the detection to be validated); as 16 is not available in the Windows version of TRF, we used 20. The minimal length of Mreps was set to 8 bp, but the length filter eliminates all detections smaller than *period *+ 9 bp, which *de facto *gives a minimal detection size of 10 bp for mononucleotides, 11 bp for dinucleotides, etc. As very long microsatellites are rare, though not absent, no maximum size was fixed in Mreps options. TRF alignment weights, Sputnik mismatch penalty, and Mreps resolution principally affect the divergence level, but this criteria is still largely unknown and no consensus or limit can be proposed at this time. We kept values advocated by developers, *i.e.*, {2,7,7} for the alignment weights of TRF, -6 for the mismatch penalty of Sputnik, and 1 for the resolution of Mreps (as resolution 0 provides only perfect detections).

### Statistical methods

The variation in length distributions between different TRF threshold score parameters was analyzed using analyses of covariance (ANCOVA) under a linear regression model [52] Type III. Detection numbers were the dependant variable (in log10), length was a covariate, and the parameter settings were included as a factor. As the distributions roughly follow a negative exponential in the window of 25–70 bp, the use of a linear regression model on the variable in log10 is appropriate. The variation in length distributions between Sputnik' validation scores was analysed using the same ANCOVA test in the range of 20–70 bp. Length comparisons between algorithms were also performed using ANCOVA tests, taking algorithms as a qualitative factor and using linear regressions. Distributions were normalized prior to analysis. Indeed, Sputnik shrinks all detections to the largest size multiple of the motif size, by discarding the incomplete end repeat. This means that all non-multiple lengths are lacking from the distributions, while multiple lengths are artificially increased. Linear regressions were performed on integer parts of the detection numbers, for the five algorithms. This critically decreases the power of the tests, especially for penta- and hexanucleotides, with regressions based on ten and eight points respectively. Comparison among species were conducted using Kruskal-Wallis tests on algorithms, for detection numbers, average lengths, and average divergences.

## Authors' contributions

S.L. collected the data, ran the comparisons and formated the results. All authors conceived the study and contributed to the discussion. All authors were equally involved in writing the manuscript.

## Supplementary Material

Additional file 1 Number of detections (log scale) with TRF in the human X chromosome as a function of length (in bp) for different alignment weights. Click here for file

Additional file 2 Number of detections per megabase, average length (bp), and average divergence (%) of detections for combinations of parameters in the human X chromosome. Click here for file

Additional file 3 Length distributions of perfect detections (log scale) for the six motif classes and the five algortihms, on the 2L chromosome of Drosophila melanogaster. Click here for file

Additional file 4 Length distributions of perfect detections (log scale) for the six motif classes and the five algortihms, on the whole genome of Neurospora crassa. Click here for file

Additional file 5 Length distributions of perfect detections (log scale) for the six motif classes and the five algortihms, on the whole genome of Saccharomyces cerevisiae. Click here for file
