# Supplementary material for: Detecting microsatellites within genomes: significant variation among algorithms
Source: BMC Bioinformatics. 2007 Apr 18;8:125. doi: 10.1186/1471-2105-8-125 (PMC1876248; doi:10.1186/1471-2105-8-125)

Additional File 1- Number of detections (log scale) with TRF in the human X chromosome as a function of length (in bp) for alignment weights {2,3,5}, {2,5,5}, {2,5,7}, and {2,7,7}. The minimum alignment score is 50, and the few detections larger than 200 bp were discarded.

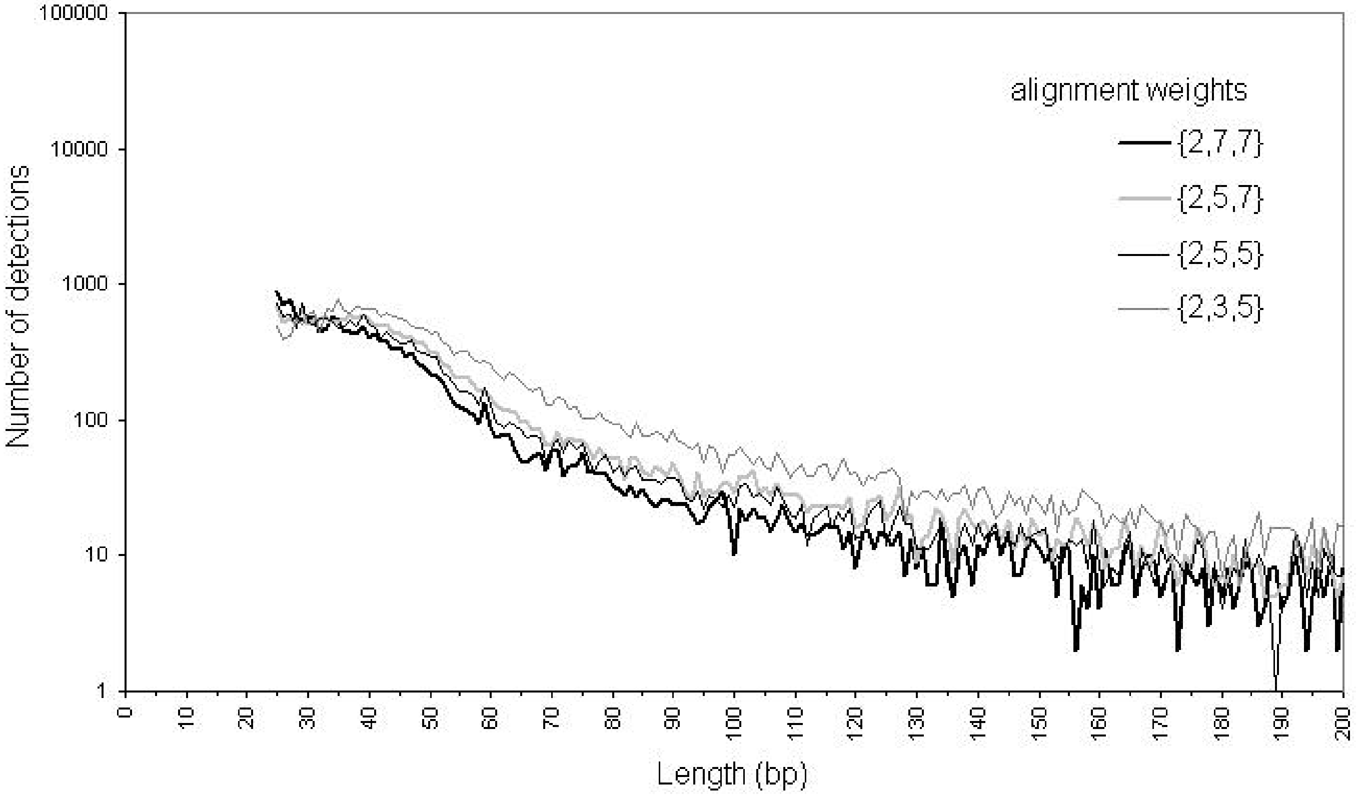

Supplement: Additional file 1 — Number of detections (log scale) with TRF in the human X chromosome as a function of length (in bp) for different alignment weights. [file 1471-2105-8-125-S1.pdf]
