# Supplementary material for: Detecting microsatellites within genomes: significant variation among algorithms
Source: BMC Bioinformatics. 2007 Apr 18;8:125. doi: 10.1186/1471-2105-8-125 (PMC1876248; doi:10.1186/1471-2105-8-125)
Supplement: Additional file 2 — Number of detections per megabase, average length (bp), and average divergence (%) of detections for combinations of parameters in the human X chromosome. [file 1471-2105-8-125-S2.pdf]

Additional File 2- Number of detections per megabase, average length (bp), and average divergence (%) of detections for combinations of parameters in the human X chromosome.

		number	length	divergence
**TRF**				
minimum score	*50*	110	64.44	3.96
	40	202	47.65	3.68
	30	458	32.14	3.21
	20	2425	16.07	1.60
align. weights				
score to 50	2,7,7	110	64.44	3.96
	2,5,7	125	73.62	6.01
	2,5,5	136	76.44	7.13
	2,3,5	177	83.30	11.31
score to 40	2,7,7	202	47.65	3.68
	2,5,7	226	53.77	5.64
	2,5,5	243	56.13	6.76
	2,3,5	316	62.30	11.31
score to 30	2,7,7	458	32.14	3.21
	2,5,7	496	36.11	5.15
	2,5,5	531	37.88	6.41
	2,3,5	716	43.02	11.70
score to 20	2,7,7	2425	16.07	1.60
	2,5,7	2466	17.56	2.82
	2,5,5	2504	18.77	3.91
	2,3,5	2782	23.06	8.43

		number	length	divergence
**Mreps**				
resolution	*1*	1368	22.96	12.39
	2	1539	28.11	18.47
	3	1636	32.21	22.15
	6	1712	39.80	26.51
**Sputnik**				
validation score				
	20	154	34.55	1.13
	15	349	25.39	1.06
	8	4273	11.23	0.48
	7	6589	9.74	0.44
mismath penalty				
	-10	6555	9.33	0.01
	-6	6589	9.74	0.44
	-5	6818	10.12	1.19

TRF alignment weights were set to {2,7,7} when varying the minimum threshold score, and alignment weights variation is presented for minimum threshold scores between 20 and 50. Mreps resolution was 1, 2, 3, and 6. Sputnik mismatch penalty was set to -6 when varying the minimum threshold score, and the minimum threshold score to 7 when varying the mismatch penalty. Match bonus and fail score were always fixed to 1 and -1, respectively. Divergence is deduced from the alignment of the detected sequence with the perfectly repeated corresponding sequence of focal consensus motif: $divergence = (substitutions + insertions + deletions)/alignment\ length)$
