# Supplementary material for: Detecting microsatellites within genomes: significant variation among algorithms
Source: BMC Bioinformatics. 2007 Apr 18;8:125. doi: 10.1186/1471-2105-8-125 (PMC1876248; doi:10.1186/1471-2105-8-125)

Additional File 4- Length distributions of perfect detections (log scale) in the whole genome of *Neurospora crassa* for the six motif classes using TRF with alignment weight {2,7,7} and minimum alignment score 20, Mreps with resolution 1, Sputnik with validation score 7 and mismatch penalty -6, STAR, and RepeatMasker.

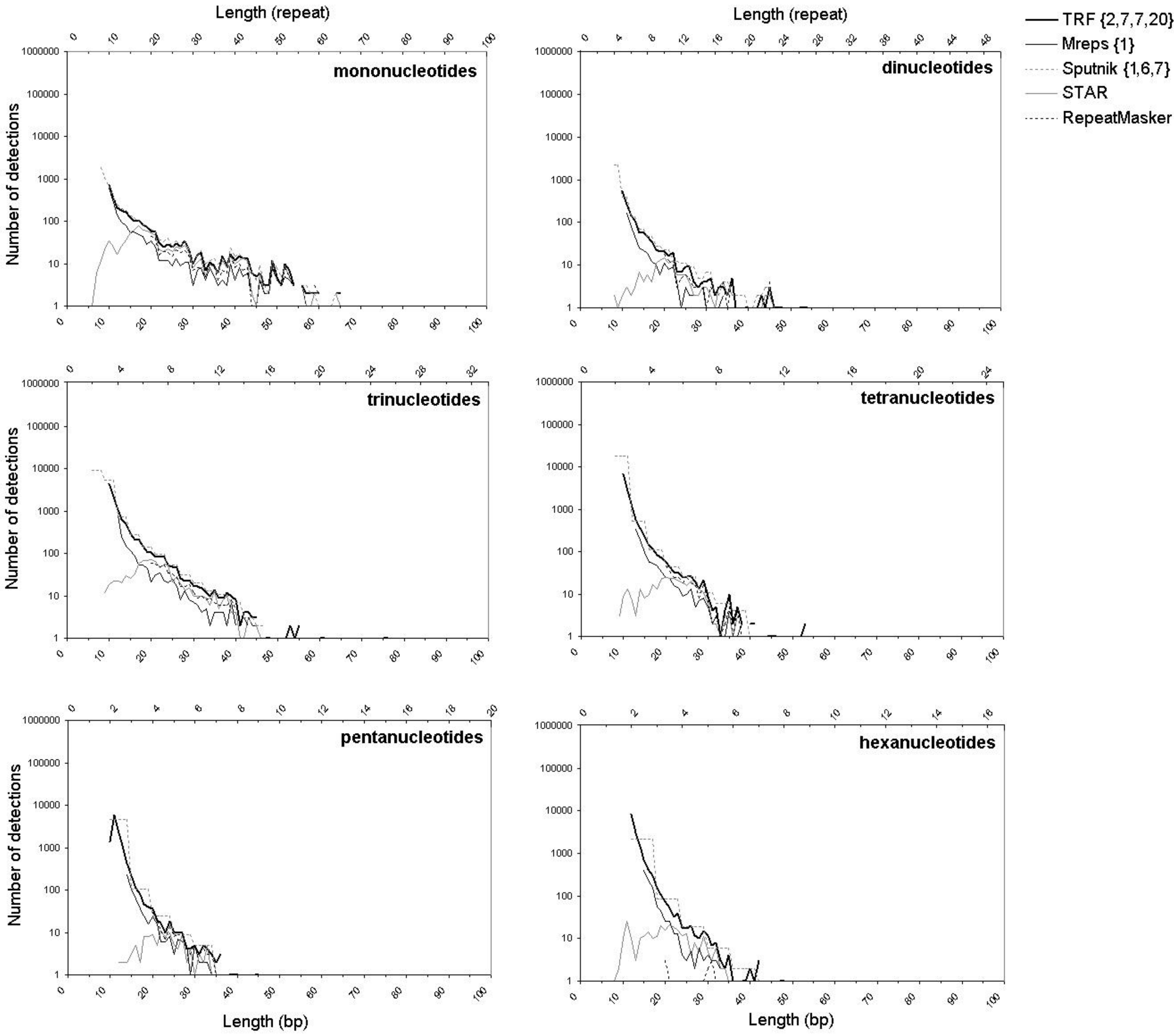

Supplement: Additional file 4 — Length distributions of perfect detections (log scale) for the six motif classes and the five algortihms, on the whole genome of Neurospora crassa. [file 1471-2105-8-125-S4.pdf]
