# Supplementary material for: Detecting microsatellites within genomes: significant variation among algorithms
Source: BMC Bioinformatics. 2007 Apr 18;8:125. doi: 10.1186/1471-2105-8-125 (PMC1876248; doi:10.1186/1471-2105-8-125)

**Additional File 5**- Length distributions of perfect detections (log scale) in the whole genome of *Saccharomyces cerevisiae* for the six motif classes using TRF with alignment weight {2,7,7} and minimum alignment score 20, Mreps with resolution 1, Sputnik with validation score 7 and mismatch penalty -6, STAR, and RepeatMasker.

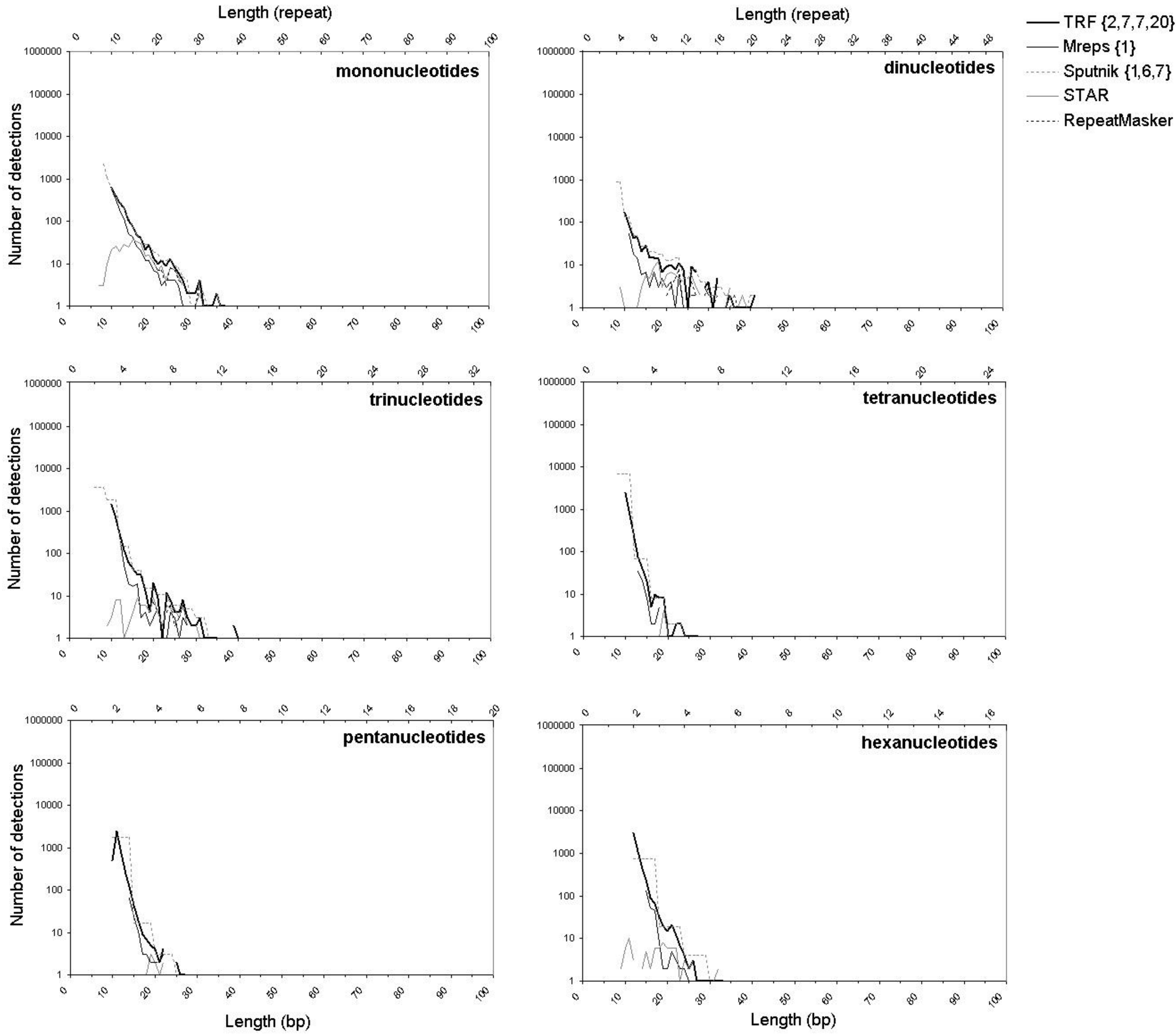

Supplement: Additional file 5 — Length distributions of perfect detections (log scale) for the six motif classes and the five algortihms, on the whole genome of Saccharomyces cerevisiae. [file 1471-2105-8-125-S5.pdf]
